# Mortality, and life expectancy in Epilepsy and Status epilepticus—current trends and future aspects

**DOI:** 10.3389/fepid.2023.1081757

**Published:** 2023-02-23

**Authors:** Eugen Trinka, Lucas J. Rainer, Claudia A. Granbichler, Georg Zimmermann, Markus Leitinger

**Affiliations:** ^1^ Department of Neurology, Christian-Doppler University Hospital, Paracelsus Medical University, Centre for Cognitive Neuroscience, Member of EpiCARE, Salzburg, Austria; ^2^ Neuroscience Institute, Christian-Doppler University Hospital, Paracelsus Medical University, Centre for Cognitive Neuroscience, Salzburg, Austria; ^3^ Institute of Public Health, Medical Decision-Making and HTA, UMIT – Private University for Health Sciences, Medical Informatics and Technology, Hall In Tyrol, Austria; ^4^ Department of Neurology, Meir Medical Center, Kfar Saba, Israel; ^5^ Team Biostatistics and Big Medical Data, IDA Lab Salzburg, Paracelsus Medical University, Salzburg, Austria; ^6^ Research and Innovation Management, Paracelsus Medical University, Salzburg, Austria

**Keywords:** seizures, death rate, standardized mortality ratio, case fatality, epilepsy

## Abstract

Patients with epilepsy carry a risk of premature death which is on average two to three times higher than in the general population. The risk of death is not homogenously distributed over all ages, etiologies, and epilepsy syndromes. People with drug resistant seizures carry the highest risk of death compared to those who are seizure free, whose risk is similar as in the general population. Most of the increased risk is directly related to the cause of epilepsy itself. Sudden unexplained death in epilepsy patients (SUDEP) is the most important cause of epilepsy-related deaths especially in the young and middle-aged groups. Population based studies with long-term follow up demonstrated that the first years after diagnosis carry the highest risk of death, while in the later years the mortality decreases. Improved seizure control and being exposed to a specialized comprehensive care centre may help to reduce the risk of death in patients with epilepsy. The mortality of status epilepticus is substantially increased with case fatality rates between 4.6% and 39%, depending on its cause and duration, and the age of the population studied. The epidemiological data on overall and cause specific mortality as well as their determinants and risk factors are critically reviewed and methodological issues pertinent to the studies on mortality of epilepsy and Status epilepticus are discussed.

## Introduction

Epilepsy is a severe neurological condition affecting approximately up to 70 million people worldwide (1–3). With an incidence of 50/100,000 patient years and a prevalence of 700/100,000, epilepsy accounts for more than 0.5% of the total global burden of disease (1). Each year, 2.4 million people are diagnosed with epilepsy, contributing to 20.6 million disability-adjusted life years lost (1).

There is a large variation in the prevalence of epilepsy in different parts of the world, especially between high- and low-income countries (4). The majority of people with epilepsy (PWE) live in low- and middle-income countries in South-East Asia, Latin America, and sub-Saharan Africa, where the rate of new cases is up to two-fold higher than that of high-income countries (1, 5). Throughout each region, lifetime prevalence rates of epilepsy are 6/1,000 population in Asia and western countries, 17.8/1,000 population in Latin America, and 15.0/1,000 population in sub-Saharan Africa (3, 6, 7). There is also a tendency towards a higher prevalence of epilepsy in rural areas than in urban areas (4, 6).

However, as many as three-quarters of people with epilepsy in low-income countries do not have access to the treatment they need, due to low availability and affordability of antiseizure medications (ASM). Additionally, misconceptions, stigma, and discrimination are greater obstacles to the well-being of people with epilepsy than lack of adequate healthcare in these areas (8).

Persons with epilepsy, who become seizure free on ASMs can almost invariably lead a normal life. The consequences of drug resistant epilepsy are the major driver on the huge burden associated with epilepsy. In drug resistant epilepsy, comorbidities are significantly increased and further complicate the management of this serious neurological condition (9). Comorbidity is defined as the presence of one or more additional conditions co-occurring with a primary condition. The term is used to describe the simultaneous presence of two chronic diseases or conditions in a patient (10). The prevalence of comorbidity increases with age, affecting more than 50% of adults with active epilepsy (11). Comorbidities are associated with several negative outcomes, including a poorer prognosis for seizure control and significantly reduced quality of life. Affective disorders, anxiety, psychosis, autism spectrum disorder and various cognitive impairments are more common in PWE than in the general population (12–17). Under-diagnosis and under-treatment of psychiatric comorbidities might result in worse seizure-control and reduced life-expectancy, with depression and anxiety even leading to self-stigmatization, suicidal ideation, and eventually suicide (18–21). Therefore, the International League Against Epilepsy has developed guidelines for treatment of depression and anxiety in epilepsy (22). Furthermore, people with epilepsy are more likely to have chronic somatic conditions such as stomach/intestinal ulcers, stroke, urinary incontinence, bowel disorders, migraine, Alzheimer disease, myocardial infarction, and chronic fatigue than the general population (11, 20), associated with a worse outcome and increased mortality (23).

Of special concern is the increased mortality rate among people with epilepsy compared to that of the general population (24, 25). Mortality is significantly higher in persons with seizures of a structural or known (symptomatic) cause, as compared to unknown (i.e., cryptogenic) or idiopathic (no structural, metabolic cause, but presumed genetic) cause; mortality is higher in children than in adults, and lower in incidence than in prevalence studies (26). The most important immediate causes of mortality include sudden unexpected death in epilepsy (SUDEP), status epilepticus (SE), injuries, and suicide. Standardized mortality ratios range from 1.6 to 3.0 in high-income countries (25) to 19.8 [95% confidence interval (CI) 9.7–45.1] in low- and middle-income countries (24). Male patients, children and adolescents, as well as patients with lack of access to health facilities show a slightly increased standardized mortality ratio (26). SUDEP among the general population of people with epilepsy reaches an incidence of 1.2 per 1,000 person-years (95% CI, 0.9–1.5), with an incidence of 1.3 (95% CI, 0.9–1.8) in adults above 50 years of age and 1.1 (95% CI, 0.5–2.3) in children under the age of 16 years (27). Major risk factors for SUDEP include the persistence of seizures, nocturnal seizures, and generalized tonic-clonic seizures (28).

To investigate mortality in epilepsy, researchers need to select a representative sample of the population with epilepsy. This can be difficult to do on a global scale, as populations with epilepsy differ in many ways (e.g., frequency of risk factors and age composition). While some factors (e.g., age) can be controlled for in the analysis, others (e.g., distribution of risk factors) cannot. There are many potential sources of data that can be used to identify people with epilepsy, including diagnostic registries at hospitals, EEG laboratories, and groups with increased risk for epilepsy (29). If these registries are thought to have identified most people with epilepsy in a study area, the study is population-based and representative of the general epilepsy population. These studies give good estimate on prevalence overall but may suffer from poor phenotyping of the seizure type, epilepsy syndrome or the causes. If hospital registries are used as the sole source of data, phenotyping and precise diagnosis is a great advantage over population-based studies, however, they are prone to a biased sample that overestimates the mortality of all people with epilepsy. Prognostic studies of mortality rates should ideally include all people newly diagnosed with epilepsy. The best way to achieve this is through incidence studies, which identify all new cases over a specified period of time. An incidence cohort will contain a higher proportion of mild epilepsy cases than a prevalence cohort (29).

In this review, we aim to update current knowledge of the methodological aspects of research on mortality in epilepsy, causes of mortality, mortality in status epilepticus, as well as to set our sights on future directions in mortality research.

## Methods

For this narrative review, data were collected from MEDLINE®/PubMed® and EMBASE® using specified search criteria (papers published in English from 1980 to 2022; International League Against Epilepsy classification papers published pre-1996 were allowed). The search terms were combinations of the following: “epilepsy” and “mortality”. We used the additional filters: “only full text”, “clinical trial”, “meta-analysis”, “randomized controlled trial”, “review”, and “systematic review”. Overall, we screened 1,042 abstracts. If a title or abstract described a high-quality article that was likely to be eligible for inclusion, the full article was obtained and assessed for relevance. Overall, 135 articles investigating the epidemiology of epilepsy, diagnosis, comorbidities and associated mortality, stigmatization, and treatment were included in this narrative review. A limitation of this approach is that the selected studies are not always comparable, with diverse methodologies and endpoints, but the mail purpose of this narrative review was to give the reader an overview on the topic.

## Methodological aspects: how do standardized mortality ratios translate into life expectancy?

Statistically, in its simplest form, mortality can be quantified by considering a subject at a given point in time as either dead or alive. Consequently, this binary variable might be analyzed using standard methods such as calculating proportions. According to the specific characteristics of the chosen statistical approach (e.g., with or without adjustment for demographic and clinical characteristics), the resulting quantities are referred to by well-established epidemiological terms, which will be outlined in the sequel along with the respective interpretations. It should be noted that these concepts may be considered as special cases within the more general framework of “survival analysis” or “time-to-event analysis” [for an overview, see, Klein & Moeschberger, 2003 (30)]. For this type of analyses, a more fine-scale quantification of mortality is used by considering the time between, e.g., diagnosis of the disease and death. Although this information is usually available, survival analysis approaches are somewhat scarce in epidemiological studies on epilepsy and only four studies addressed this specifically (14, 31–33). At least partially, this might be because in epidemiology, subjects are often observed over quite long time periods, which often results in survival patterns exhibiting complex functional forms. While the survival analysis toolbox also contains flexible modelling approaches, the standard quantities based on proportions might still be preferable, due to their straightforward interpretation. However, especially for life expectancy calculations and comparisons, bridging the gap between the two approaches might yield new insights, which is briefly outlined at the end of this section.

In the sequel, it is assumed that mortality is quantified using the binary indicator “death” vs. “alive”. The proportion of deaths divided by the size of the population of interest (e.g., the population within a specific geographic region) is called *(crude) mortality rate*. If the population of interest is restricted to subjects with a particular disease (e.g., epilepsy), the term *case fatality rate* is used instead. Frequently, a specific time point / interval is chosen for these calculations (e.g., 1-year mortality). Further subtle yet important aspects regarding the definition of the “population of interest” (e.g., how to quantify the “subjects at risk”) are not discussed in detail here. In the sequel, for ease of presentation, the term “mortality rate” is used, although the methods might be applied to case fatality rates as well.

Frequently, the probability of dying is influenced by several demographic and clinical characteristics. For example, age and sex are considered as important predictors of death. Consequently, especially the comparison of crude rates between different epidemiological studies might be questionable, due to potential differences in the demographic and clinical characteristics of the underlying respective populations. Therefore, a *standardization* of the rates, which comprises the following steps, may solve this problem (34):

(1) The first step in all standardization approaches is to calculate mortality rates for specific subgroups that are formed according to those demographic and clinical variables which are considered as important predictors of death. For example, groups according to age decades (i.e., interval 1: 1–10, interval 2: 11–20, interval 3: 21–30, etc.) might be formed, and subsequently, the interval-specific mortality rates *m_i_* = *d_i_ / n_i_* for interval number *i* are used as age-group-specific estimates.
(2) Now, a straightforward idea would be to apply *direct standardization*, which is based on the following idea: Assume that the probability of dying is 30 percent. How many deaths are expected in a population of size 1000, then? Intuitively, one would expect 300 deaths (i.e., 1,000 times 0.3). Indeed, this is also sensible from a mathematical viewpoint, and applying this idea to the age-specific rates from step (1) yields the age-group-specific (expected) number of deaths *W_i_ = N_i_ × m_i_*, where *N_i_* denotes the number of subjects in age group i in the so-called *reference population*. The reference population might be one of the available well-established standard populations (https://seer.cancer.gov/stdpopulations/, accessed 2022-09-11), or more generally any population that is appropriate with respect to the epidemiological research question.
(3) Finally, the sum of the quantities *W_i_* is calculated and divided by the total size of the reference population (i.e., the sum of the *N_i_*'s), in order to obtain a proportion again. So, eventually, the *age-adjusted mortality rate* is defined as
  $$\frac{\sum_{i=1}^{k}N_{i}\;\times \;m_{i}\;}{N}$$

Thereby, *N_i_* denotes the number of subjects in age group *i* in the reference population, and *N* denotes their sum. The quantities *m_i_* are the age-group-specific mortality rates in age group *i* in the study cohort, and *k* denotes the number of age groups.

By contrast, an *indirect standardization* approach would replace the calculations outlined above by the following procedure, which tackles the problem in the opposite way (i.e., using the mortality data from the reference population as the “ground truth” for subsequently calculating the expected mortality in the study cohort):

(1) The age-group-specific mortality rates in the reference population are calculated at first.
(2) Then, these rates are multiplied with the numbers of subjects within the specific age groups in the study cohort, in order to obtain an age-group-specific expected number of deaths in the study cohort.
(3) The expected numbers of death from step (2) are summed up, in order to obtain the total number of deaths in the study cohort that would be expected if the mortality rates from the reference population were also true for the study cohort. Finally, the *total number of actually observed deaths in the study cohort* is often divided by that aforementioned *total expected number of deaths*, in order to obtain the *standardized mortality ratio (SMR)*.

Complementing the—adjusted or unadjusted—rates, the *estimation of life expectancy* might be an additional yet less frequently used means of quantifying mortality. The *life expectancy at a certain age* (also called *mean residual life at a given time point*) is informally defined as the expected number of years a subject has left to live. It is obvious from this (informal) definition that a higher level of detail regarding the data—instead of only considering “alive” vs. “death” at a prespecified single time point—is required here, which naturally gives rise to adopting methods from survival analysis, as briefly outlined at the beginning of this section. Indeed, several proposals for life expectancy estimation and comparison have been published (e.g., (32)], and methodological research on the corresponding challenges has been quite vivid recently [e.g. (35, 36)].

## Mortality in epilepsy

Looking at mortality data we have to differentiate between the different cohorts. Especially the geographic location of the study influences the numbers, with significantly higher rates in low- and middle-income countries (LMIC) compared to high income countries (HIC) (24, 25). Further, the setting of the cohort is of significance, whether it is population-based or hospital-based, as selection-biases might be introduced. The strength of population-based cohorts is the representative sample ideally including all patients with known or diagnosed epilepsy in a geographic region. The weakness is often an uncertain diagnosis or no verification of the diagnosis at al. For instance, in a landmark study in the UK 1,091 patients were recruited in a prospective population-based cohort, but later epilepsy was confirmed in only 564 (37). Other weaknesses are the usually shorter follow up in population-based cohorts, and finally the regional differences in a given country cannot be addressed, when comparing to a national standard reference population (21, 38). The major strength of hospital-based cohorts is the diagnostic accuracy, the detailed identification of etiology (21, 38). Among other health care restrictions many people in LMIC do not receive appropriate ASMs and epilepsy surgery is hugely underutilized. The so-called treatment gap is defined as “the number of people with active epilepsy not on treatment (diagnostic and therapeutic) or on inadequate treatment, expressed as a percentage of the total number with active epilepsy (39, 40).” The treatment gap was estimated in a systematic review covering Africa, Asia, and Latin America to be as high as 56% of PWE, who do not receive adequate epilepsy treatment (5). In LMICs the availability of basic public health services as well as access to specialized health care for epilepsy varies considerably between LMICs as well as within LMICs between urban areas and rural parts of the respective country. (https://apps.who.int/iris/bitstream/handle/10665/170250/9789240694439_eng.pdf;jsessionid=2248AD37F7F5B801D04BC57A04B5F280?sequence=1, accessed on 2023-01-29) This has significant impact of mortality in LMIC, but mortality data on population-based samples reporting SMRs and life expectancy calculations from LMICs still sparse (7). In LMIC it is almost impossible to ascertain the precise number of deaths because incident studies are difficult, death certificates are notoriously unreliable (not quite different in HICs), autopsy rate is very low, and the high migration rate make large population based prospective studies almost impossible (41). Methods to assess excess mortality on LMICs are often door-to door surveys or selected cohorts.

Standardized mortality rates (SMR) in population-based studies of HICs lie consistently between two and three, with only few outliers, whereas most of the studies reporting SMRs in LMICs vary between three and five. (Table 1). Numbers in hospital-based studies and other selected cohorts are similar, although with more variance (Table 2).

a. Population based cohorts
b. Hospital based cohorts
c. Risk factors for Mortality in Epilepsies

**Table 1:** Overall mortality in epilepsy patients—selected population-based cohorts.

Author	Country	Cohort	SMR^a^ (95% CI)
Population-based studies, all ages			
Lhatoo, 2001 (42)	UK	Incidence	2.1 (1.8–2.4)
Lindsten, 2000 (43)	Schweden	Incidence	2.3 (1.9–2.6)
Hauser, 1980 (44)	USA	Incidence	1.7 (1.1–2.3)
Benn, 2008 (45)	USA	Incidence	3.0 (2.5–3.7)
Cockerell, 1997 (37)	UK	Incidence	2.1 (1.7–2.6)
Morgan, 2002 (46)	UK	Prevalence	1.6 (1.2–2.2)
Olafsson, 1998 (47)	Island	Incidence	2.6 (1.8–3.5)
Neligan, 2011 (48)	UK	Incidence	2.6 (1.8–3.5)
Rakitin, 2011 (49)	Estland	Incidence	2.1 (1.8–2.4)
Ding, 2013 (50)	China	Prevalence	2.9 (2.6–3.4)
Mu, 2011 (51)	China	Prevalence	4.92 (4.0–6.1)
Dreier, 2022 (14)	Denmark	Prevalence	3.0 (3.0–3.1)–2.7 (2.7–2.8)
Banerjee, 2010 (52)	India, Kolkata	Prevalence	2.58 (1.50–4.13)
Population-based studies, children			
Sillanpaa, 2010 (53)	Finland	Incidence and prevalence	6.4 (5.9–7.0)
Nickels, 2012 (54)	USA	Incidence	6.9
Camfield, 2002 (55)	Canada	Incidence	7.5 (4.4–13.0)

CI, confidence interval; SMR, standardized mortality ratio; RR, relative risk.

^a^ Unless otherwise indicated.

**Table 2:** Overall mortality in epilepsy patients—selected hospital-based cohorts and other special cohorts.

Author	Country	Cohort	SMR^a^ (95% CI)
Hospital-based cohorts—all ages			
Nilsson, 1997 (56)	Sweden	Hospitalized patients	3.6 (3.5–3.7)
Mohanraj, 2006 (57)	UK	Reference center	1.4 (1.2–1.7)
Trinka, 2013 (21)	Austria	Reference center	2.2 (2.0–2.4)
Granbichler, 2015 (58)	Austria	Reference center	1.7 (1.6–1.9)
Chen, 2016 (59)	Hong Kong	Hospitalized patients	5.09 (4.88–5.31)
Chang, 2012 (60)	Taiwan	Reference center	2.5 (2.2–2.8)
Chen, 2005 (61)	Taiwan	Reference center	3.47 (2.46–4.91)
Tran, 2008 (62)	China	Communitiy-based phenobarbital program	Case fatality 11%
Carpio, 1999 (63)	Ecuador	Reference center	6.3 (2.0–10.0)
Carpio, 2005 (41)	Mali	Door-to-door survey	Case fatality 16%
Carpio, 2005 (41)	Martinique	Hospitalized patients	4.25
Bharucha, 1988 (64)	India (Parsi)	Door-to-door survey	0.76 (0.51–1.01)
Bharucha, 1988 (64)	India (Vasai)	Door-to-door survey	7.81
Hospital-based cohorts—children			
Callenbach, 2001 (65)	Netherlands	Incidence	7.0 (2.4–11.5)
Berg, 2004 (66)	USA	Incidence	7.5 (4.4–13.0)

CI, confidence interval; SMR, standardized mortality ratio; RR, relative risk.

^a^ Unless otherwise indicated.

### Etiology

Etiology of the epilepsy is one of the most important of those factors. Those that suffer from neurological disorders as an underlying cause of the epilepsy, especially when the central nervous system is involved or a learning disorder is present, mortality rates are higher. This is especially true when a brain tumor is present with an up to 50-fold increase in mortality (37, 42, 44, 48, 54, 55, 65–71). Including these patient groups in calculations of mortality in epilepsy is questionable, as the underlying condition determines the chances for premature death rather than the epilepsy itself. Therefore, many studies exclude brain tumors in their estimates. But even then, SMR values remain elevated in all studies, with 2.2–4.3 (21, 37, 42, 44, 45, 47–49).

For cryptogenic and idiopathic epilepsies only marginally increased or normal SMRs have been found, at 0.9–2.1 (37, 42, 44, 45, 47–49).

### Age and sex

Even though mortality is increased in all age-groups, a certain age-correlation can be shown, and younger patients show higher values than older age groups (37, 43, 44, 53, 55, 56, 65, 66, 72). A Swedish cohort reported an SMR of 9.5 and 10.7 for age-groups 15 to 39 and 40 to 59, respectively, while it was only non-significantly elevated at 1.3 in those over 80 (56). Similarly, a study from the United Kingdom reported and SMR of 7.6 in those aged 0 to 49, and 2.6 in those over 80 years of age (37). The same was true in an American cohort with 8.5 for ages 0 to 24, and 1.4 for those above 74 (44).

The influence of sex, however, is more controversially discussed. While mortality is elevated for both sexes, some studies have shown higher values for men than women, while others could not confirm this (21, 43, 45, 46, 56). Only one study from Island of an incidence cohort with unprovoked seizures could show that there was a significant difference in SMR between women [0.8 (CI, 95% 0.4–1.5)] and men [2.3 (CI, 95% 1.6–3.1)] (73). Also an older study from the US states an SMR of 1.6 in women, and 2.1 in men, however, confidence intervals were not provided and we therefore do not know if this difference is significant (44).

### Duration of disease

The more time passes after a first epilepsy diagnosis, the lower the likelihood of premature death. The highest mortality is within the first two years after diagnosis (9, 21, 58). Weather SMR remains elevated at all years later is unclear, but several studies have shown an increased SMR even 20 years following initial diagnosis (21, 42, 44, 47). Epilepsy etiology then seems to be the most important influential factor, with structural etiology showing much higher values in the first years following diagnosis that then decrease significantly until they reach values comparable to those of epilepsies without structural changes (21, 31, 47).

### Seizure types

Even though data is limited on this factor, it appears that tonic-clonic seizure (GTCS), primary or secondary, have the most significant influence on mortality. There are only three studies that investigated this: a Swedish cohort that found an SMR of 3.9 for GTCS, and 2.1 for other focal onset seizures (43), a more recent study from Estonia, where GTCS had an SMR of 2.7 compared to 1.5 for focal onset seizures (49), and an American study that reports an SMR of only 1.3 for GTCS, while focal onset seizure showed an SMR of 1.8. However, in the latter study no detailed information on seizure type is available (45).

### Life expectancy

There are only very few studies on life expectancy in epilepsy (14, 31, 33, 35). In 1974, a subgroup analysis of a population-based study in Warsaw reported a life expectancy of 12.5 years following the diagnosis of epilepsy, which was on average 20 years shorter than that of the general population in Poland at that time (33). Data from the UK National General Practice Study of Epilepsy were used to calculate the life expectancy using the parametric Weibull model (31), a well-recognized statistical technique for exploring the relationship between the survival of a patient, and several explanatory variables. From 564 persons with epilepsy, the life expectancy was reduced by up to 2 years in those with idiopathic and cryptogenic epilepsy, and up to 10 years in those with symptomatic epilepsy, compared to the general population of UK. There was also a decrease in years of life lost with increasing time from epilepsy diagnosis. Unfortunately, the numbers were too small for further subgroup analysis; moreover, the assumptions of the statistical model were rather restrictive insofar that they did not allow for increased life expectancies of the patients. Apart from that, the variability of the life expectancy estimates (e.g., confidence intervals) was not provided, thus rendering the interpretation in comparison with other studies difficult. In addition, the life expectancy was compared with the general UK population neglecting thereby geographic differences of baseline mortality (31). A more recent study analysed the life expectancy of newly diagnosed persons with epilepsy in a large cohort with well-defined adult epilepsy, by comparing life expectancy with that of the general population living in the same geographic area in Tyrol, Austria (35). The authors applied a Weibull regression model using gender, age at diagnosis, epilepsy etiology, and year of diagnosis as covariates at time of epilepsy diagnosis, and 5, 10, 15, and 20 years after diagnosis. Yet, no *a priori* restrictions were set regarding the mortality rates and life expectancies of the patients. This work confirmed a reduced life expectancy in symptomatic epilepsy until the 1990s, mainly during the first years following diagnosis up to 7.4 years, but on the other hand most subgroups did not show changes in life expectancy compared to the control population. Unexpectedly during the 2000s, life expectancy was even prolonged for those with cryptogenic epilepsy independent of the time since diagnosis (35). These findings cannot be explained easily but are most likely to improved epilepsy care and early identification of drug resistant epilepsy in a comprehensive care centre (74, 75).

## Cause-specific mortality

Proportional mortality was reported in several hospital- and population-based studies (21, 44, 56, 58, 76–80). Neoplasms account for 5%–26%, and pneumonias for 5%–25% of deaths (77, 78). The largest percentage of deaths is caused by pneumonias in cohorts of institutionalized patients (12%–25%) (77, 78, 80), while the population-based studies and the large Swedish study reported clearly smaller percentages (up to 5.5%) yielding SMRs between 4.0 and 7.2 (44, 56, 76). Deaths from cerebrovascular disease are different, in that the population-based studies showed clearly higher percentages, namely 12%–17% (44, 76), than did the studies of institutionalized patients with only 5%–6% of deaths due to cerebrovascular diseases (77, 80). One exception is the Tyrolean mortality study that, similar to the population-based studies, shows a high percentage of cerebrovascular deaths, namely 15% (21, 79). Accidents are given as the cause of death in 1%–16% of mortalities (21, 33, 44, 76–79, 81). Here, too, the studies conducted in institutionalized patients show clearly higher figures than do the population-based studies (1%–6%) (44, 76). The findings for suicide are particularly divergent, namely up to 21% of deaths reported by some studies (33, 58, 77, 78, 81, 82), and less than 1% of deaths reported by the large population-based studies in Rochester and the United Kingdom (44, 76). Since proportional mortality does not permit any conclusions to be drawn on elevated mortality in an actual as compared to a standard population, the SMR must be calculated for the particular cause of death. In summary, these studies show a consistently elevated SMR for pneumonia (SMR 3.5–10.3), tumors (SMR 1.5–4.8) and cerebrovascular disease (SMR 1.8–5.3) (21, 44, 56, 58, 76–80). Elevated SMRs were also reported for accidents and other external causes of death (SMR 2.4–5.6) (21, 44, 56, 70, 79, 80). The results with regard to suicide are contradictory, as already mentioned: while the population-based studies showed no elevated SMR (44, 76), the other studies showed a clearly elevated SMR ranging from 3.5 to 5.4 (56, 80). A review with special emphasis on the medical risks of epilepsy including physical injuries, mortality and traffic accidents has been published (83).

Wannamaker, who analyzed the literature from 1910 to 1974, reported that 42.7% of deaths are directly related to epilepsy (84). Causes of death directly related to epilepsy are status epilepticus, accidents occurring during an epileptic seizure, bolus death from aspiration and sudden unexpected death in epilepsy patients (SUDEP) (85). Studies conducted in institutionalized patients in Finland and the United Kingdom found occurrences directly related to epilepsy to be the most common cause of death, namely 19%–31% (77, 78, 80). In children with epilepsy, causes of death directly related to epilepsy, such as SUDEP, drowning, injuries, or status epilepticus, are also very common, namely 22%–45% (86, 87). By contrast, the two population-based studies from Rochester (USA) and the United Kingdom show clearly smaller percentages, namely 3%–4% [(44, 76), *p*. 198]. Differences in case ascertainment, classification criteria and the length of the observation period might play a role here. Identification PWE through population databases or insurance data may lack diagnostic accuracy of epilepsy and also of causes of death (21, 25, 37); and compared to hospital-based cohorts epilepsy may no longer be the primary diagnosis any more, especially when competing diagnoses, such as stroke or dementia appear in the records. In addition, the rate of autopsy may influence the causes, attributed to the death of a PWE. Standardized reporting on death certificates is highly recommended for future research (88).

## Mortality in status epilepticus

Status epilepticus (SE) is associated with a significant mortality and accounts for ∼10% of epilepsy-related deaths (89). Two recent reviews summarize all population-based studies on status epilepticus and the respective case fatalities in adults (Table 3) (38, 90).

**Table 3:** Population based studies of adults with status epilepticus (38). (Reproduced with permission from Leitinger and Trinka et al., Epilepsy & Behavior, Elsevier).

Author	History of epilepsy	Acute symptomatic	Remote symptomatic	Pro-gressive	Defined electro-clinical syndrome	Crypto-genic	Febrile status^j^	Case fatality, %
Logroscino (91)	n.a.	53.8^d^	46.2^d,l^				excl.	24^k^
Hesdorffer (92)	46	50.3	19.6	8.5	13.6^m^		8	n.a.
Dham (93)	1.8–7	n.a.	n.a.	n.a.	n.a.	n.a.	n.a.	9.2
DeLorenzo (94)	42	n.a.	24	n.a.	n.a.	n.a.	n.a.	22^k^
Wu (95)	n.a.	n.a.	n.a.	n.a.	n.a.	n.a.	n.a.	10.7
Betjemann (96)	n.a.	n.a.	n.a.	n.a.	n.a.	n.a.	n.a.	n.a.
Jallon (97)	32.8	50.8	26.2^l^				23.0	6.6
Coeytaux (98)	43	62.7	18.6	9.8	2.9	5.8	n.a.	7.6
Knake (99)	33^b^	>33^e^	62.7^e^	12.0^h^	n.a.	8.7	0	9.3^k^
Vignatelli (100)	39	34^f^	34	11	7^m^		0	39^k^
Vignatelli (101)	40.7	29.6^g^	25.9	11.1	n.a.	7.4	0	7^k^
Govoni (102)	40	25.0	45.0	15	15^m^		n.a.	5
Strzelczyk (103)	44.6	24.8	n.r.	n.r.	n.r.	4.3	n.r.	14.8
Leitinger (104)	40.7	36.2	46.6	14.0	1.4	1.8	0	16.3
Rodin (105)	43.9	26.8	48.8	7.3	n.a.	17.1	0	24.4
Kantanen (106)	17.5	41.6	45.3	12.4	n.a.	10.9	0	9.0^k^
Nazerian (107)	57.6	68.7	37.8	27.3	n.a.	6.1	0	13.1
Ong (108)	n.a.	n.a.	n.a.	n.a.	n.a.	n.a.	n.a.	8.8
Tiamkao (109)	n.a.	n.a.	n.a.	n.a.	n.a.	n.a.	0	8.4
Tiamkao (110)	1.2	n.a.	n.a.	n.a.	n.a.	n.a.	0	14.5
Bhalla (111)	0	35.4	44.6	3.1^h^	n.a.	16.9	n.a.	18.5
Bergin (112)	60.6^c^	43.3	43.6	5.2	3.5	17.7	21.0	4.6^k^
Vijiala (113)	24.3	41.1	40.2	13.1	0	5.6	n.a.	7.8

N.A., not available.

^a^ proposal for definition and classification of SE by ILAE 2015; ^b^primary service area; ^c^calculated per status epilepticus episodes and not per patients; ^d^1975–1984; ^e^ in most cases more than one factor; ^f^multifactorial: additional 14%; ^g^multifactorial: additional 25.9%; ^h^tumors; ^j^only in children; ^k^case fatality at 30 days, otherwise in hospital; ^l^number represents the sum of remote symptomatic, progressive, defined electroclinical syndromes, and cryptogenic; ^m^number represents the sum of defined electroclinical syndromes, and cryptogenic.

Mortality in SE is associated with drug resistance (104): Refractory (RSE) and Super-Refractory SE (SRSE) are associated with a death rate of up to 39.5% and 37.5%, respectively (103, 106, 114–116). While discharge mortality in nonrefractory SE is 9.6% % (103). In a large case series of new onset refractory SE (NORSE), mortality was 23% (6/26) (117).

In a pediatric population of 100 children, mortality was 10% in SRSE, 1.9% in RSE and 0% in non-refractory SE compared to 3.6% in the total population (118). A retrospective study on 109 patients hospitalized in the neuro-intensice care units for SE revealed intubation, hypotension and a low GCS at presentation as risk factors for an increased mortality (119).

Recurrent SE showed a mortality at 30 days of 2% compared to the initial episode of SE with 22% (120). Within those who relapsed within six months, mortality after the second event at 30 days was 24% compared to the group with relapse after 6 months with 27% (120). In the US, readmission rate at 30 days was 15% after investigation of a nationwide database with 42,232 adults most commonly (45.1%) due to seizures (121). After multivariable analysis, independent risk factors were intracranial hemorrhage (odds 1.56, 95% CI, 1.12–2.18), psychosis, diabetes mellitus, chronic kidney or liver disease, more than three comorbidities, length of stay more than 4 days during index hospitalization, and discharge to a skilled nursing facility (121).

In a 10-year study on 14,487 deaths associated with status epilepticus, cardiac arrest was the cause of death at autopsy in 21.3% (122). Women were at a lower risk of myocardial infarction (odds ratio 0.55, 95% confidence interval 0.51–0.61), patients 45 years or elder had a higher risk of developing myocardial infarction, arrhythmia, heart failure or cardiac arrest compared with people with epilepsy, unspecified convulsions, febrile convulsions, or posttraumatic seizures (122).

In an elderly population in Taiwan the in-hospital mortality of 77 patients with *de novo* CSE was 38.9% (123). Multivariable analysis revealed the presence of comorbidities (OR 0.23, 95% CI, 0.0059–0.879), low Glasgow Coma Scale (OR 0.045, CI, 0.013–0.160) and *de novo* SE (OR 0.093, CI, 0.017–0.503) as parameters significantly related to mortality. A recent systematic review about the elderly showed that mortality was reported 71 out of 85 identified studies (124). In another systematic review, mortality within the elderly with SE was highest short-term 22%–38% and long-term 82% which resulted in a standardized mortality ratio, i.e., the relative risk of mortality compared with the general population, was 2.2 (95% CI, 1.6–2.9) in those aged over 65 years (125).

The influence of treatment on the mortality, especially when using anesthetic drugs has been discussed intensively in the recent years. Ferlisi and Shorvon (126) reviewed 159 publications with reported outcome data in 1,061 patients. The long-term outcome was death in 35%, but remarkably higher death rates during treatment were found with phenobarbital/thiopental with 19% compared to 2% with midazolam and 8% with propofol. In a 6-year cohort study on 171 patients with SE, 37% were treated with intravenous anesthetic drugs, the mortality was 18%. It has to be considered, that the side effects of the treatment may significantly contribute to the mortality of RSE and SRSE.

**Figure 1:**
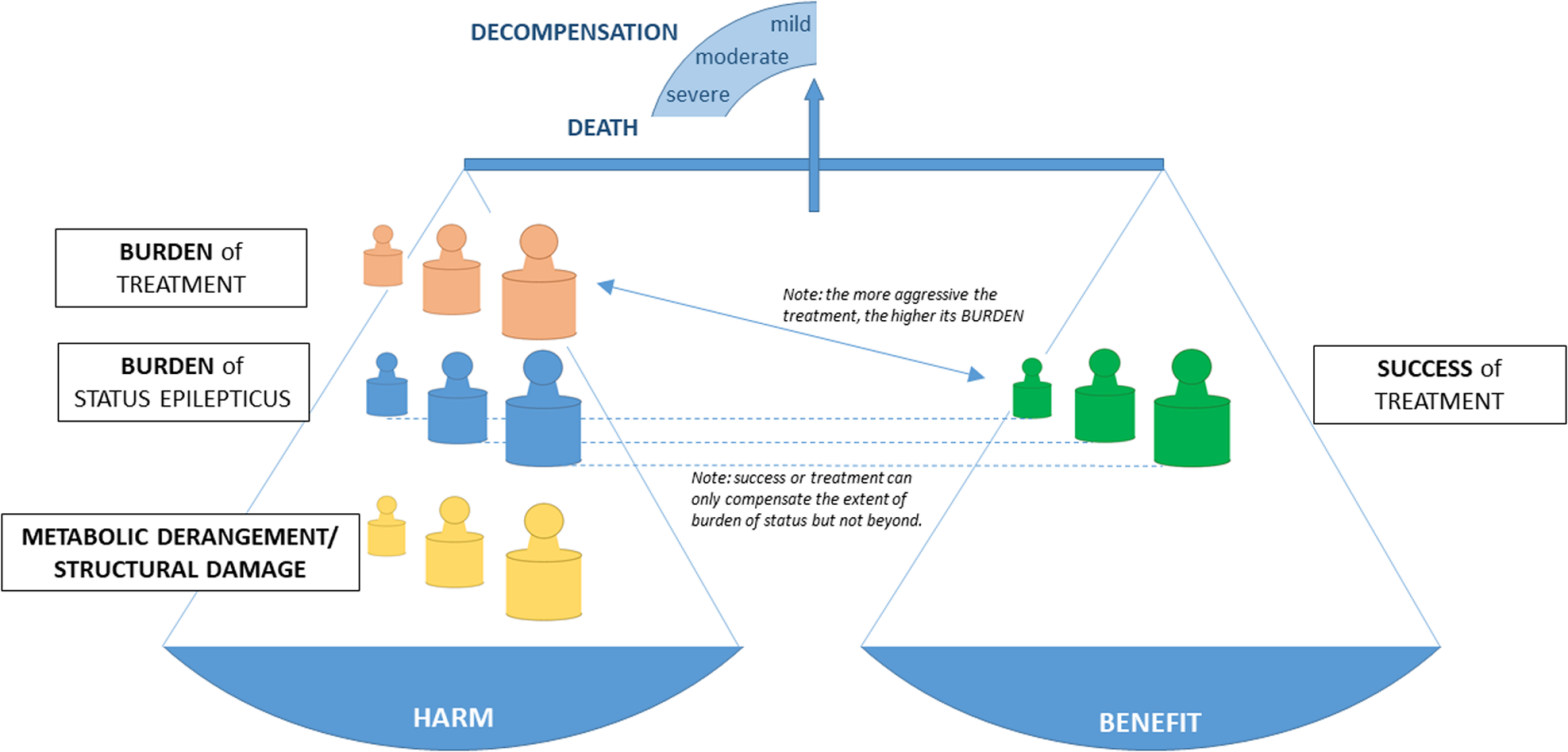
The impact of burden model integrates the structural damage and metabolic derangement, the burden of status, the success of treatment, the burden of treatment, and the impact of these burdens in an individual patient [modified from Trinka and Leitinger, 2022 (127)].

Recently, the impact-of-burden-model was created to provide a framework to implement the various factors (i.e., etiology of SE in form of structural damage and metabolic derangement, burden of status epilepticus, and burden of treatment) a patient with SE is exposed to (Figure 1) (127). In short, this framework shows that the benefit from treatment is the net gain of success of treatment (i.e., reduction of status burden) and burden of treatment. The impact of these burdens depends on the amount of functional reserve or decompensation which are determined by structural pathologies or metabolic derangements and further by comorbidities, age, and several other factors. It becomes apparent that individuals need a treatment adapted to their functional reserve to prevent decompensation and that studies need appropriate outcome parameters. Mortality should only be used in populations with high structural/ metabolic burden, e.g., subarachnoid hemorrhage or cerebral hypoxia due to cardiac arrest. Septicemia or moderate metabolic derangements require functional outcome parameters as readout as people will mostly survive at least on a short time basis.

Among epidemiological studies on SE there is a substantial heterogeneity making comparisons between studies difficult. The most important influential factors are: (i) the age profile of the study population and the reference population used for adjustment, (ii) the time to establish the diagnosis of SE, (iii) the inclusion of only first episodes of SE or also of recurrent episodes, (iv) the spectrum of etiologies including a prior history of epilepsy, (v) case ascertainment by ICD-codes or of file-based diagnosis of SE, (vi) the inclusion of both adults and children in one study, (vii) and the timepoint of outcome, i.e., at hospital discharge or at 30 days (38). Overall, there is a lack of recent data on the epidemiology, mortality, and healthcare burden associated with SE using the 2015 ILAE definition of SE. Available data suggest a high burden of illness and mortality, which is associated with, age, etiology, duration, and drug resistance in SE.

## What has changed in the past decades?

**Figure 2:**
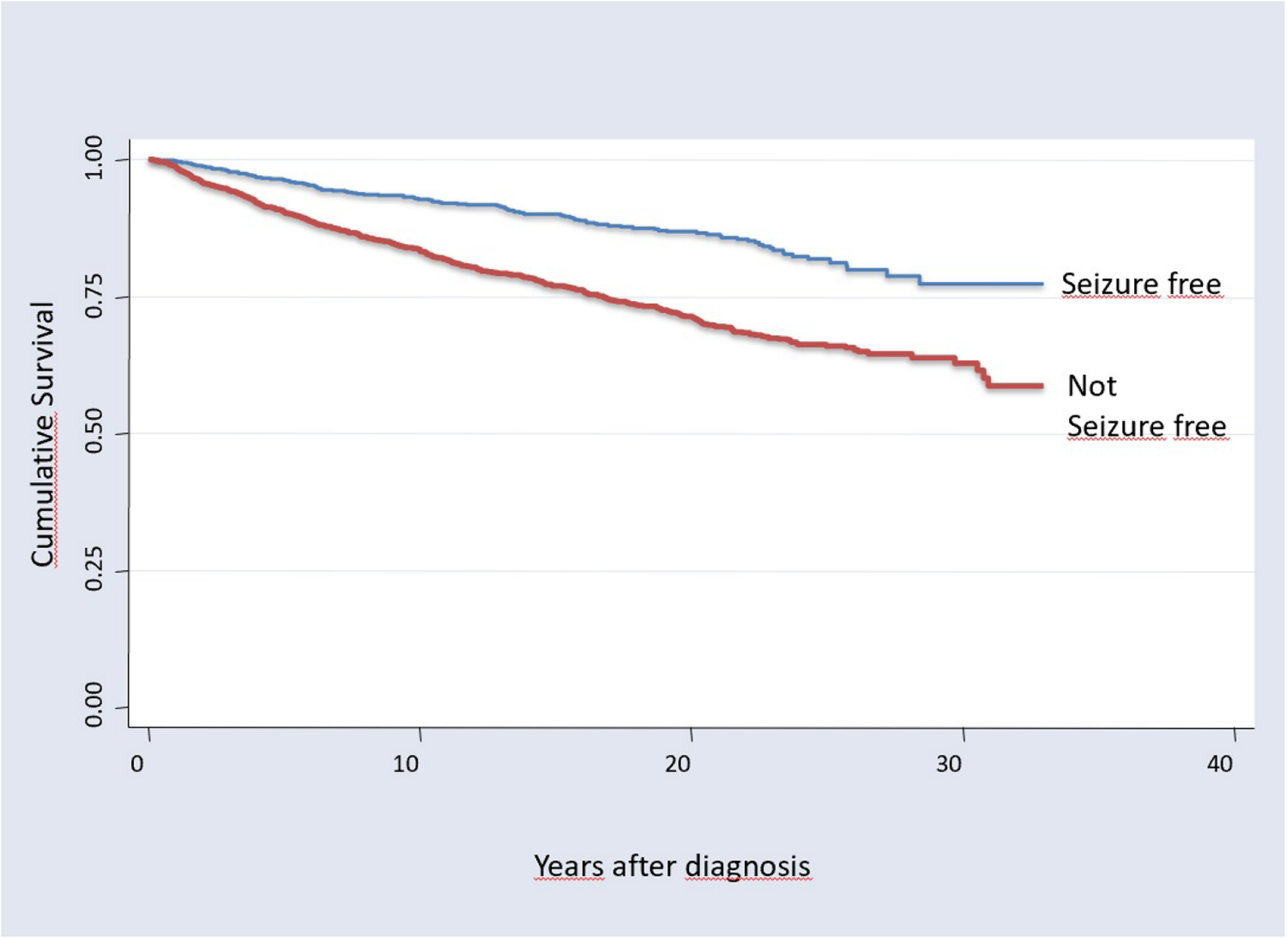
Kaplan maier survival curves of 3,334 patients in tyrol, Austria, with more than 30 years observation; overall 48,595 person years. Seizure free patients have a significant better overall survival (log rank test *χ* = 80,3, *p* < 0.0001), [from Trinka, 2005 (79)].

Mortality studies on epilepsy have been carried out systematically since the 1950ties. Astonishingly there was the general view until recently, that mortality did not change over time, despite major advancements in treatment including effective ASMs and successful epilepsy surgery. O’Donoghue and Sander used data from the Chalfont Centre for Epilepsy, UK, a residential centre for people with epilepsy, and determined the SMR in the Chalfont population for each 5-year epoch from 1896 to 1965 (128). The authors concluded *“that an excess mortality has been associated with chronic epilepsy for 100 years despite major changes in treatment.”*. Another more recent systematic review on nine population based studies performed between 1974 and 2006 found SMRs between 1.6 and 5.3 without evidence that the *“SMR or the mortality rate of people with epilepsy has changed significantly over time”* (129). Several studies demonstrated that the excess mortality is caused by the drug resistant epilepsy patients, and seizure free patients have no increase in SMR (21, 57, 79) (Figure 2).

A time trend analysis of the Tyrolean Epilepsy Cohort (21, 58, 74, 79) found a decrease of mortality since 1980: The overall SMR decreased from 3.0 (95% CI, 2.1–4.3) in 1980–1989, to 2.7 (95% CI, 2.0–3.5) in 1990–1999 and to 1.4 (95% CI, 1.0–2.0) in 2000–2007 (74). The reason for this decline is not easy to explain, but the early recognition of drug resistant epilepsy and the introduction of epilepsy surgery in this centre are the most plausible explanations (74). More recently a large study from the Comprehensive Calgary Epilepsy Programme, Alberta, Canada found a clear significant association of levels of specialized epilepsy care and excess mortality in patients with epilepsy (75). Among the 23 653 incident cases the overall standardized mortality rate was 7.2%. It was 9.4%for those receiving nonspecialist care, 5.6%for those seen by a neurologist, and 2.8%for those seen in the Comprehensive Calgary Epilepsy Programme. The hazard ratio (HR) of mortality was significantly lower in those receiving neurologist (HR, 0.85; 95% CI, 0.77–0.93) and Comprehensive Calgary Epilepsy Programme (HR, 0.49; 95% CI, 0.38–0.62) care (75). This study clearly showed that specialized care of epilepsy patients saves lives.

In Status epilepticus, similarly a decrease of deaths from SE was observed: Neligan and Walker analysed SE mortality data from 2001 to 2013, and compared it to annual age group populations for England and Wales: All epilepsy deaths significantly decreased (Spearman's q −0.733, *p* = 0.004), which is predominantly due to a decrease in SE deaths (Spearman's q −0.917, *p* < 0.001) (89). Along the same lines, the number of patients admitted to CCU for SE in the UK were rising three-fold from the early 2000 years to the early 2010 years whereas acute hospital mortality was decreasing in 35,595 CCU cases, especially in neurological critical care units from 8.1% to 4.4% in the same period of time (130). In sum, these finding supports the hypothesis that the policy of early and aggressive treatment of SE (Trinka and Leininger, Continuum 2022) may be improving the outcome, and most importantly decrease mortality.

## Future directions

After more than 50 years of modern public health research, it has been shown that the premature death of patients with epilepsies, more specifically drug resistant epilepsies, and SE can be reduced by adequate treatments and comprehensive care. But still according to the WHO Epilepsy Report (https://apps.who.int/gb/ebwha/pdf_files/EB146/B146_12-en.pdf/, accessed 2023-01-29, and https://www.who.int/news/item/28-04-2022-draft-intersectoral-global-action-plan-on-epilepsy-and-other-neurological-disorders-2022-2031, accessed 2023-01-29), epilepsies rank fifth among all neurological causes for disability-adjusted life years (DALYs) (131). Worldwide, an estimated 125,000 people die each year due to epilepsy (131). The risk of premature mortality for people with epilepsy is estimated at now is three times that of the general population (25). In some low-resource settings around the world, this risk may be increased up to seven-fold (24, 132). However, up to 70% of people with epilepsy could become seizure-free following an accurate diagnosis and use of cost-effective and commonly available, ASMs. The research gap in mortality of epilepsy and status epilepticus between HIC and LMICs is huge and more research in LMICs is urgently needed. Moreover, the socioeconomic determinants of mortality are often neglected or understudies. Modifiable risk factors are well known, with access to appropriate treatments as the most important one. Ideally future research on mortality will involve more population-based incident cohorts from LMIC and collect data on comorbidities, and specific causes of death, as well as socioeconomic data. The reference population for such studies should take regional differences of mortality into equation.

The recently approved WHO Intersectorial Global Action plan for Epilepsies and other Neurological Disorders (133) calls for a multi-stakeholder approach driven at the national and local level to reduce the treatment gap, stigma, and aim for 70% of the people with epilepsy to be seizure free. There is hope that these measures will dramatically reduce the premature mortality in the future.
